# Misfolding and Amyloid Aggregation of Apomyoglobin

**DOI:** 10.3390/ijms140714287

**Published:** 2013-07-09

**Authors:** Clara Iannuzzi, Rosa Maritato, Gaetano Irace, Ivana Sirangelo

**Affiliations:** Department of Biochemistry, Biophysics and General Pathology, Second University of Naples, Via De Crecchio7, Naples 80138, Italy; E-Mails: clara.iannuzzi@unina2.it (C.I.); rosa.maritato@unina2.it (R.M.); gaetano.irace@unina2.it (G.I.)

**Keywords:** apomyoglobin folding, apomyoglobin misfolding, amyloid aggregation

## Abstract

Apomyoglobin is an excellent example of a monomeric all α-helical globular protein whose folding pathway has been extensively studied and well characterized. Structural perturbation induced by denaturants or high temperature as well as amino acid substitution have been described to induce misfolding and, in some cases, aggregation. In this article, we review the molecular mechanism of the aggregation process through which a misfolded form of a mutated apomyoglobin aggregates at physiological pH and room temperature forming an amyloid fibril. The results are compared with data showing that either amyloid or aggregate formation occurs under particular denaturing conditions or upon cleavage of the residues corresponding to the *C*-terminal helix of apomyoglobin. The results are discussed in terms of the sequence regions that are more important than others in determining the amyloid aggregation process.

## 1. Introduction

Amyloidosis is an emerging category of diseases characterized by the extracellular accumulation of protein aggregates in body organs or tissues. These disorders include cerebral conditions such as Alzheimer’s disease, Parkinson’s disease, and Creutzfeldt-Jakob disease, and also a series of systemic amyloidoses in which amyloid deposition occurs in a wider variety of organs within the body [1,2]. Although the first cases of amyloidosis were described over 300 years ago, it is only within the past 20 years that the specific chemical composition and structure of amyloid have been understood. More than 20 different kinds of amyloidosis are known currently [3]. All have in common the presence of insoluble protein aggregates, generally termed “amyloid” [1,4,5], that share several physicochemical features: a fibrillar morphology, a predominantly β-sheet secondary structure, birefringence upon staining with the dye Congo red, insolubility in common solvents and detergents, and protease resistance. The amino acid sequence and the native structure of the proteins associated with amyloid diseases have been found to be highly variable, but structural studies have revealed that amyloid fibrils from different sources share a common ultra-structure. Amyloid fibrils are typically straight and unbranched and are formed from an assembly of protofilaments 2–5 nm wide. X-ray diffraction analysis has indicated a characteristic structure, the β-cross motif, in which the polypeptide chains form β-strands oriented perpendicular to the long axis of the fibril, and β-sheets propagating in the fibril direction [6–10].

**Figure 1:**
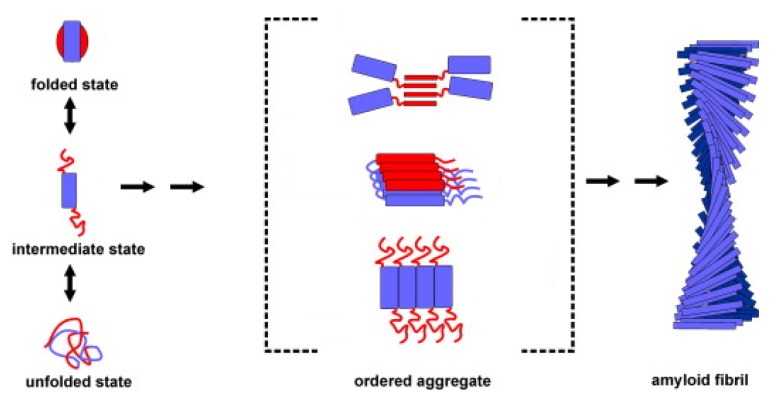
Association of two or more non-native peptide/protein molecules forming highly ordered, fibrillar aggregates.

Aggregating proteins are molecules with a misfolded structure, *i.e.*, which results from the inability of the protein to fold correctly into its functionally active conformation or to maintain it [11]. There are various causes of protein misfolding that can lead to amyloid formation. For example, in the absence of chaperones, certain proteins fail to achieve their native state and may associate with each other. Misfolding can also occur when a protein is subjected to particular conditions, such as extremes of heat or pH. Moreover, misfolding is often associated with specific mutations that reduce the stability of the folded state [12–16]. Recent studies have allowed three major factors to be identified as important parameters in the conversion of the partially or totally unfolded state of a protein into aggregates. These are high hydrophobicity, high propensity to convert from α-helical to β-sheet structure, and low net charge [17–23]. Protein destabilization favors the formation of partially unfolded conformations that are highly prone to aggregation [24]. In these states, many of the hydrophobic residues and amide and carboxyl groups are exposed to the solvent and, therefore, susceptible to associate with each other resulting in the formation of protein aggregates. The association of two or more non-native peptide/protein molecules, largely driven by the hydrophobic interactions, gives rise to the formation of amorphous structures with a granular morphology as well as of highly ordered, fibrillar aggregates (Figure 1). Fibrillar aggregates may also originate from the assembly of soluble protein molecules in their native or native-like state, *i.e.*, a conformation with only few minor structural modifications compared to the native form of the protein [25–28].

The ability to form amyloid fibrils is not a peculiar property of the relatively few amino acid sequences associated with specific diseases, but it is a generic phenomenon of a polypeptide chain. In fact, a considerable number of proteins, not involved in any amyloid disease, including those adopting full α-helical structures under native conditions, have been shown to form amyloid fibrils *in vitro* [29–32]. The ability of polypeptide chains to aggregate into morphologically similar amyloid-like fibrils, independently of the amino acid composition and sequence of the precursor proteins, has suggested that this process may be rationalized in terms of relatively simple, universally valid physicochemical principles. Such a property arises from the intrinsic tendency of polypeptide chains to self-organize into polymeric assemblies, which are stabilized by inter-molecular hydrogen bonds established between the peptide bonds of parallel or anti-parallel polypeptide stretches in a β-strand conformation. In this respect, natural proteins can be regarded as amino acid polymers selected by evolution so that their amino acid sequences are optimized to disfavor aggregation whilst favoring folding into compact, yet not rigid, states. This is mainly due to tertiary interactions among the side chains that shield not only the hydrophobic core but also the peptide backbone [33]. Conversely, protein aggregation into amyloid polymers, which are mainly stabilized by secondary interactions, can be considered the result of the emergence, under non-natural conditions, of the intrinsic primordial tendency of the peptide backbone to give secondary intermolecular interactions [1,33,34]. Thus, protein folding and protein aggregation are considered distinct but competing processes, and the environmental conditions dictate which one is favored for a given polypeptide chain [35].

Amyloid fibril formation *in vitro* is preceded by the formation of metastable, non-fibrillar forms often referred to as prefibrillar aggregates. These species have the appearance of spherical particles of 2–5 nm in diameter [36–38]. Prefibrillar precursors are often associated into bead-like chains or annular rings such as “doughnut” shaped structures [39–43]. Such assemblies appear to be precursors of longer protofilaments and mature fibrils that appear only after longer time.

**Figure 2:**
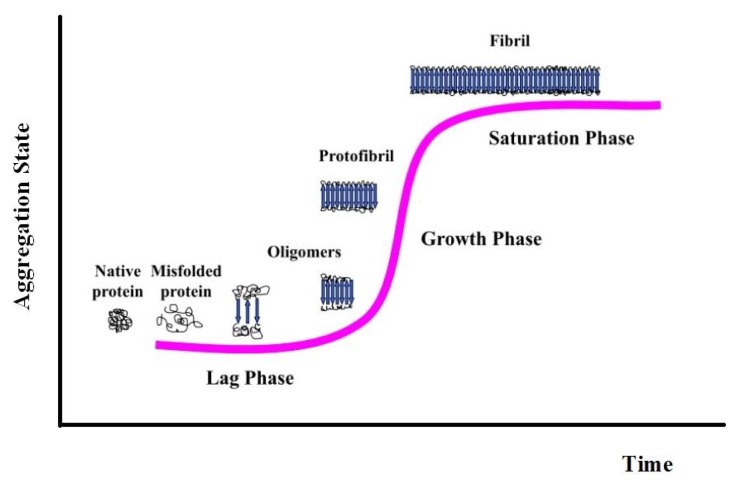
Nucleation-dependent fibril formation process.

Fibril formation is a nucleation-dependent polymerization process which can be simply described by a sigmoid curve, indicative of a three-stage process consisting of protein misfolding, nucleation, and fibril elongation [44] (Figure 2). In the first phase, called “lag phase”, soluble protein species, usually monomers, associate to form nuclei and the transition to oligomeric species with β-sheet conformation occurs. The protein precursor is responsible for the aggregation process via a variety of predisposing events to realize its fibrillogenic potential. Nucleus formation requires a series of association steps of monomers, which are thermodynamically unfavorable, representing the rate-limiting step. The second phase is the “exponential phase” or “growth phase.” Once a nucleus has been formed, further addition of monomers to the nucleus becomes thermodynamically favorable, resulting in rapid extension of fibrillar structures *in vitro* [45]. The path of fibril formation begins with pre-fibrillar kinetic precursors, collectively indicated as soluble, ordered aggregates. These species are oligomeric to an extent that exceeds the oligomer state required for normal function of the protein, and contain non-covalently-bound repeating units, which appear as globules 2.5–5.0 nm in diameter or larger. In the extension process, a key role is played by forces common to all proteins, without any meaningful dependence on the specific peptide sequence: hydrophobic interactions, backbone hydrogen bonding, stacking interactions. At the end of the second phase, larger ordered structures, termed protofibrils because of their intrinsic fibrillar structure [38,46], are formed. They represent the initial stable elements in the fibril formation pathway. Fibrils are completely formed during the third phase, or “saturation phase” [47]. There are two possible fibril growth mechanisms: β-sheet elongation, in which the fibril grows by adding individual peptides to the end of each β-sheet, and lateral addition, in which the fibril grows by adding an already-formed β-sheet to its side. Both mechanisms seem to play an equally significant initial role in fibril development. It has also been suggested that, consequently, two distinct phases in human fibrillogenesis can take place, where lateral growth of oligomers is followed by longitudinal growth into mature fibrils [48].

The nucleation-polymerization model has been validated by the observation that fibril extension kinetics accelerated by the addition of preformed fibrils, *i.e.*, by a seeding effect [49]. In conclusion, amyloid aggregation occurs via multiple pathways [50,51] that are populated by distinct aggregated species, including soluble oligomers, protofibrils and annular species. However, it is still under debate whether these species are ‘on pathway’ intermediates for fibril formation or represent ‘off pathway’ species that may serve as a buffer monomer concentration or otherwise [52].

## 2. Apomyoglobin Folding

Proteins spontaneously fold from randomly unfolded conformations to biologically active structures in a hierarchical manner, with secondary structure preceding tertiary structure formation [53]. Secondary structure is primarily stabilized by hydrogen bonds between the amide groups of amino acids that are close in sequence [54,55], whereas tertiary structure is stabilized by hydrophobic interactions among side-chains of more distant segments of the chain [56,57]. This is supported by the observation that fluctuating elements of secondary structure often persist under denaturing conditions where the chain is disordered and devoid of specific tertiary interactions. Under physiological conditions, hydrophobic interactions among non-polar side-chains favor collapse of hydrogen-bonded secondary structure elements into a compact conformation [58]. In this respect, folding is usually envisaged as the convergence of an ensemble of disordered conformations, *i.e.*, the unfolded state, toward lower-energy partially folded compact structures from which the biologically active protein is obtained [59].

**Figure 3:**
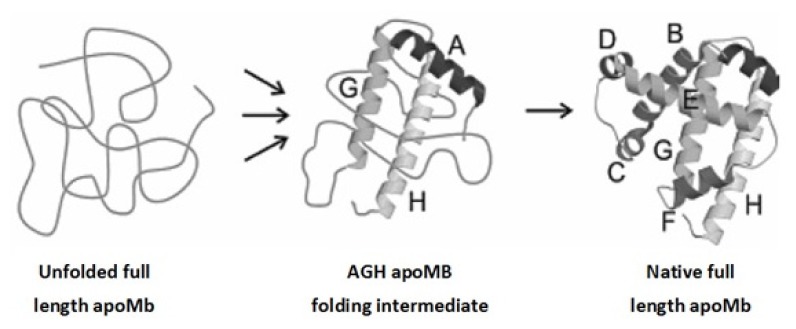
Schematic illustration of apomyoglobin (apoMb) folding.

Apomyoglobin, *i.e.*, heme-free myoglobin, is a small, alpha-helical protein that contains two highly conserved tryptophanyl residues located at positions 7 and 14 in the *N*-terminal region of the molecule. The folding of this protein is known to proceed through compact intermediates that have been detected in both kinetic and equilibrium experiments [60–65]. In most of these intermediates, A, G, and H helices are folded and sterically oriented as in the native AGH subdomain, whereas the remainder of the molecule seems to be unordered (Figure 3). Uzawa *et al*. [66] presented evidence that the high level of helical structure in the earliest compact intermediate suggests the presence of helical regions outside the A, G, and H helix subdomain. Furthermore, the same authors supposed that the increases in helix content observed in the subsequent folding stages are probably due to an increase in the length of the pre-existing helices. The additional, secondary and tertiary structure modules are subsequently formed [67]. Once folding has occurred, the heme binds to the crevice formed essentially by E and F helices.

## 3. Apomyoglobin Misfolding and Amyloid Formation

In amyloid fibrils the main chain dominates the structure and the side-chains are incorporated in the most favorable manner consistent with this requirement. By contrast, in the evolved globular structures, the overall fold is determined by the close-packing of the side-chains, and the polypeptide backbone is incorporated in the most favorable manner. Globular proteins may then have evolved features to prevent aggregation by selecting and preserving key residues that interfere with the establishment of the interactions in the polypeptide backbone that would lead to aggregation. In this respect, the tryptophanyl residues located in the A helix of myoglobin seem to play such a crucial role in preventing the main chain from taking over the network of interactions that stabilizes the native three-dimensional structure. The simultaneous replacement of both indole residues determines a deviation from the correct folding pathway leading to protein aggregation and amyloid formation even under physiological conditions [68,69], whereas the presence of at least one of the two residues is required for the formation of the correct tertiary key interactions necessary for the formation of a native-like fold [70]. On the contrary, wild-type apomyoglobin forms amyloid fibrils only under stress conditions that favor the association of unfolded polypeptide segments [71,72].

**Figure 4:**
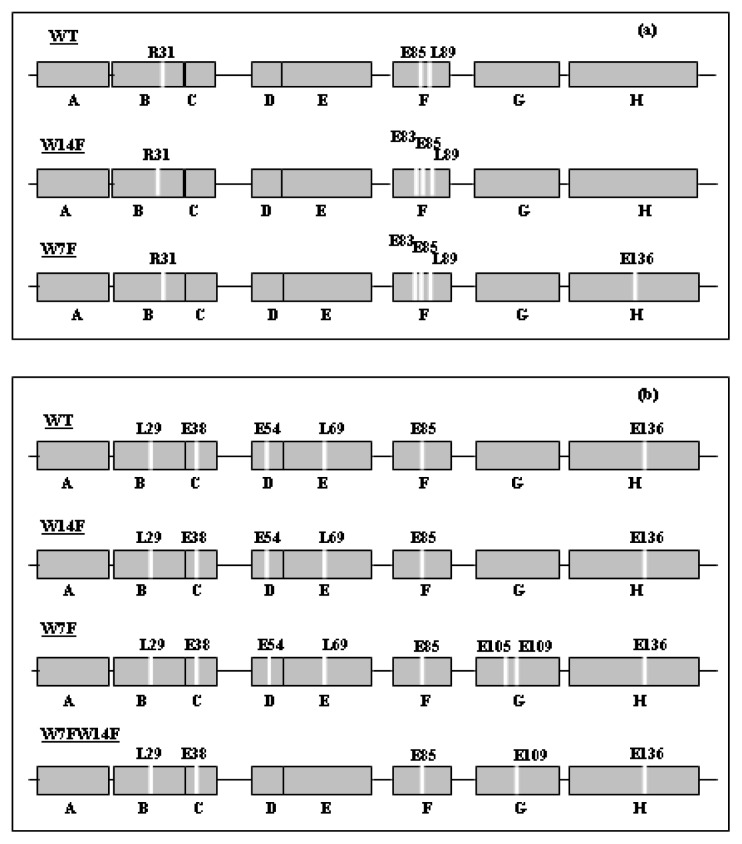
Preferential cleavage sites pattern observed in W7FW14F, wild type, W7F, and W14F apomyoglobins at pH 7.0 (**a**) and in wild type, W7F, W14F, and W7FW14F apomyoglobins at pH 4.0 (**b**) (Data from [73]).

The role played by tryptophanyl residues in driving the folding process of apomyoglobin has been recently investigated by examining three mutated proteins, *i.e.*, the single W→F mutants, W7F and W14F apomyoglobin, and the amyloid-forming double W→F mutant, W7FW14F apomyoglobin [73]. The effects caused by W→F substitutions on the structure of the native and partially unfolded state of apomyoglobin were investigated by far UV circular dichroism and limited proteolysis both at neutral and acidic pH (pH 4.0). At the latter pH value, apomyoglobin adopts the compact, partially folded state [60–65]. Particular attention was devoted to the conformational and dynamic properties of this state because of its similarity to that detected during the kinetics of refolding and, thus, representative of the early organized structure from which the native fold originates. The secondary structure compositions are displayed in Table 1, the proteolysis results are shown in Figure 4.

The lower α-helical content of both the native and partially folded state of single tryptophan-containing apomyoglobins compared to that of wild-type protein clearly indicates that each single-tryptophanyl substitution at either position 7 or 14 affects the protein secondary structure. In particular, the folding intermediate of W7F mutant, very similar to that of the amyloidogenic mutant, contains less α-helical structure and more β-content than wild type, thus indicating that the secondary structural organization of the folded portion (AGH subdomain) of the compact intermediate changes because of the substitution of the indole residue at position 7. Chow *et al*. [75] reported that the 1–36 *N*-terminal fragment of wild-type apomyoglobin displays a high level of β-structure and forms macroscopic aggregates when the pH becomes closer to neutrality. Both observations suggest that the tryptophanyl substitution at position 7 could cause an increased propensity of the *N*-terminal region to form a β-structure in the intact protein, confirm this suggestion. Infusini *et al*. [73] used some of the online available predictors, such as TANGO, PASTA, and Zyaggregator, to evaluate whether changes in the properties of the sequence are sufficient to explain these observations or, instead, structural modifications of the amyloidogenic double mutant need to be invoked. They found not only that the single substitutions W7F and W14F increase the β-aggregation propensity of segment 6–15 but also that their simultaneous occurrence has a much larger effect, a four times increase. However, even other regions other than the *N*-terminus showed a comparatively higher level of β-aggregation propensity [73].

Complementary proteolysis experiments carried out on the equilibrium intermediate formed at pH 4.0 (Figure 4) revealed few but significant differences between W7FW14F and wild-type apomyoglobin, that could be related to a different organization of the AGH core in the corresponding molten globule intermediate. In particular, in the double mutant, the region corresponding to D and E helices is protected against protease activity, while the G helix is exposed. The pattern of cleavage sites of W14F is identical to wild type apomyoglobin, with all the helices, except A and G, accessible to proteases, whereas in W7F the G helix is also accessible. Recent studies on permuted mutants [76] have indicated that a correct folding of the AGH core helps to constrain the fluctuation of the polypeptide backbone in the CDEF subdomain. The reciprocal influences of mutations in A helix on E helix have been reported by Nishimura *et al*. [77], who suggested that docking of the E helix onto the AGH core is one of the crucial steps of the apomyoglobin folding pathway, with E helix folding and packing occurring, especially when the A helix is already folded. More recently, Nishimura *et al*. [78] further confirmed that the instability at the *N*-terminus of apomyoglobin contributes to the energetic frustration of folding by preventing docking and stabilization of the E helix. In this respect, the protection of L69 in the double mutant suggests that packing of the E helix onto the AGH core mediated by this residue does not occur and, therefore, these regions may become available for amyloid aggregation. This is further corroborated by the picture of the fibril structure, revealed from proteolytic experiments, indicating that A, B, and E helices and part of D and G helices are protected from protease action and, thus, involved in the fibril core [73]. This result confirmed the most relevant aspect evidenced by the prediction analysis that the β-aggregating sequences involved in the formation of the fibril core correspond to the protein regions having the higher propensity to form an amyloid structure. Moreover, the 1–119 fragment of apomyoglobin obtained by removing the *C*-terminal segment forms water-soluble aggregates of low oligomeric state with up to 18% non-native β-strand secondary structure. The soluble apoMb119 aggregates are not fibrillogenic and do not display any significant thioflavin T binding at approximately neutral pH [75].

In conclusion, the W→F substitution at position 7 changes the secondary and tertiary organization of the AGH subdomain. However, this single mutation alone is not able to alter the productive folding pathway of W7F apomyoglobin that remains monomeric and soluble at pH 7.0 with an overall three-dimensional structure very similar to wild type, although with a significantly increased accessibility to H helix. The single substitution at position 14 has a less marked effect on the secondary structure of the compact intermediate state populated at pH 4.0. Although less marked, the reorganization of the secondary structure induced by W14F substitution increases the local flexibility of the *N*-terminal region [73,79]. When the two mutations occur together, their synergic effect determines an uncorrected pairing of the E helix on the pre-existing substructure, making the formation of the network of hydrogen bonds of the polypeptide backbone overcome the correct establishment of the tertiary interactions. This conclusion is sustained by the finding that A, B, E, and part of D and G helices participate in the formation of the amyloid fibril core. This further confirms that the mutations introduced in the *N*-terminal region are responsible not only for the increased propensity to aggregate but also for perturbing other molecular regions, especially the E and G helices, and involving them in fibril formation and elongation, with prediction analysis consistently evidencing that the regions with an intrinsic high β-aggregation propensity are α-helical structured and buried in the natively folded structure [73,79]. The simultaneous tryptophanyl replacement not only introduces structural distortion but also increases the overall flexibility of the molecule, favoring local unfolding and uncorrected helix pairings. The emerging picture is that mutated apomyoglobin forms misfolded early states with an increased propensity to form β-strands.

The mechanism described above for fibril formation from amyloidogenic apomyoglobin is substantially different from that proposed by Fandrich *et al*. [71,80] for wild type apomyoglobin. These authors reported that this protein forms amyloid fibril when incubated at 65 °C and at pH 9.0 for 24 h. The extent of fibril assembly was found to be correlated with the extent of denaturation; moreover, β-sheet-containing monomeric intermediates were not observed under the conditions of fibril formation. Because native myoglobin possesses 78% α-helical structure and because amyloid fibrils are always associated with β-sheet structure, more than 30% of the fibrillar β-sheets must be constructed from residues that are in α-helices in the folded protein. To enable fibril formation, these structural elements must first undergo unfolding. It is not known which helices might be involved in the transition from the soluble state to the β-sheet-rich amyloid fibril. However, peptide fragment corresponding to the G-helix or to the *N* terminus of myoglobin are known to form species with extensive β-structure [75,81,82]. The potential of peptide fragment corresponding to the G-helix to form amyloid structures is interesting, because the G-helix represents a very stable element of secondary structure of the globular apomyoglobin. Similarly, the *N*-terminal fragment 1–29 of apomyoglobin was found highly prone to form amyloid-like fibrils on reducing the pH from neutrality to 2.0. The aggregation properties of fragment 1–29 were rationalized by considering that protonation of the six negative moieties (two Asp, three Glu and the *C* terminus) of the peptide at low pH strongly reduces the electrostatic repulsion between the various peptide molecules, thus facilitating their association and stabilizing the resulting fibrils. More recently, it became clear that apomyoglobin can adopt two well-defined structural conformations at pH 9: below 55 °C, the helix-rich native-like structure α, and above 55 °C, the cross-β structure. The transition occurs because of the unfolding of helical structures that allows the neighbouring strands to interact with each other forming the cross-β-structure [83].

## 4. Conclusions and Perspectives

The increase in life expectancy has led to the emergence of a series of age-related disorders that pose novel challenges to modern society. Neurodegenerative disorders, including Alzheimer’s, Parkinson’s, and Amyotrophic Lateral Sclerosis are debilitating and so far incurable disorders that demand intensive research. In these diseases, misfolding, aggregation, and precipitation of proteins seems to be directly related to neurotoxicity. Environmental and genetic factors are known to be involved in amyloid aggregation process, but the mechanism by which it occurs is poorly understood. Although the W7FW14F apomyoglobin mutant is unrelated to any human disease, it is a suitable model for amyloid aggregation studies because it rapidly aggregates under physiological conditions (pH 7.0 at room temperature), forming oligomeric species that slowly convert into protofibrils and mature amyloid fibrils. The conversion of oligomeric species into mature amyloid fibrils is due to a conformational re-organization which results in the formation of the beta-cross structure. This involves not only the *N*-terminal region but also the molecular regions which form the binding site for the prosthetic group in the native globular state. The results reported in this review indicates that point mutations occurring in certain region of protein molecules may induce crucial perturbation of other, sterically related molecular regions, involving them in beta-cross structure formation. The identification of the molecular regions susceptible to amyloid aggregation is important both for rationalizing the effects of sequence changes on the protein aggregation and for the development of strategies targeted to combat diseases associated with amyloid formation. The accurate knowledge of the molecular mechanism through which amyloid is formed will certainly help to find molecules that are able to intercept the misfolded protein molecules thus preventing protein oligomerization.

## Figures and Tables

**Table 1:** Percent content of secondary structure of wild type and mutant apomyoglobin at neutral and acidic pH (Data from [73,74]).

pH 7.0	wt	W7F	W14	W7FW14F
α	0.65	0.52	0.56	0.56
β	0.04	0.09	0.07	0.09
Turn	0.09	0.14	0.12	0.14
Unordered	0.22	0.25	0.25	0.21

**pH 4.0**	**wt**	**W7F**	**W14**	**W7FW14F**

α	0.46	0.26	0.37	0.28
β	0.10	0.23	0.13	0.22
Turn	0.17	0.21	0.21	0.22
Unordered	0.27	0.30	0.29	0.28
